# 

**Published:** 1923-03

**Authors:** 


### HOSPITAL and HEALTH

REVIEW

*Established 1886* *No.* 1860. *Vol. LXXII.*

*New Series.* Vol. II. No. 18 *March* 1923 Price Sixpence

CONTENTS.

| | PAGE |
|---|---|
| The Conquest of Disease | 127 |
| "Drugs of Addiction" | 128 |
| Notes and Comments—The New Housing Programme—Politics and House Building—Röntgen and Nordau—Sir William Treloar—Co-ordination of Relief—Disposal of Refuse—Prevention of Fog—Salving the Clown—Who Shall Survive ?—Tuberculosis Statistics—Accidents and Hurry—The Diploma in Public Health—The Care of the Teeth—The Illegitimate Child—The Medical Officer's Yearly Opportunity | 129 |
| The Lady Almoner. By Joan Kennedy. | 133 |
| Tuberculosis and Housing in the West Riding | 135 |
| Combating Venereal Diseases | 136 |
| The Hospital Saving Scheme—Provincial Success and London Promise | 137 |
| Relics of Jenner | 138 |
| The Virus of Influenza | 138 |
| A Fair Chance for the Panel System. By R. W. Harris. | 139 |
| National Health Insurance Notes | 140 |
| Hospital Progress and Finance | 141 |
| Opening of Maudsley Hospital—A New Departure in the Treatment of Insanity | 142 |
| A Hospital Social Evening | 144 |
| Physically Defective Children | 145 |
| The Hospital and Nursing Exhibition | 145 |
| Correspondence | 146 |
| Reviews of Books | 148 |
| Public Health in Parliament | 152 |
| Recent Appointments | 152 |
| Government Publications | 152 |
| Echoes of the Month | 153 |
| Hospital and Health News | 155 |

